# Effectivity of ILF Neurofeedback on Autism Spectrum Disorder—A Case Study

**DOI:** 10.3389/fnhum.2022.892296

**Published:** 2022-06-09

**Authors:** Alexandra Rauter, Horst Schneider, Wolfgang Prinz

**Affiliations:** ^1^Psychological Practice, Graz, Austria; ^2^Medical Scientific Research, BEE Medic GmbH, Singen, Germany; ^3^Medical Practice, Biel/Bienne, Switzerland

**Keywords:** Othmer method, autism spectrum disorder (ASD), electroencephalogram (EEG), infra-low frequency (ILF) neurofeedback, optimal response frequency (ORF), symptom tracking, symptom severity scale

## Abstract

Autism spectrum disorder (ASD) is a neural and mental developmental disorder that impacts brain connectivity and information processing. Although application of the infra-low frequency (ILF) neurofeedback procedure has been shown to lead to significant changes in functional connectivity in multiple areas and neuronal networks of the brain, rather limited data are available in the literature for the efficacy of this technique in a therapeutic context to treat ASD. Here we present the case study of a 5-year-old boy with ASD, who received a treatment of 26 sessions of ILF neurofeedback over a 6-month period. A systematic and quantitative tracking of core ASD symptoms in several categories was used to document behavioral changes over time. The ILF neurofeedback intervention decreased the average symptom severity of every category to a remarkable degree, with the strongest effect (80 and 77% mean severity reduction) for physical and sleep symptoms and the lowest influence on behavioral symptoms (15% mean severity reduction). This case study is representative of clinical experience, and thus shows that ILF neurofeedback is a practical and effective therapeutic instrument to treat ASD in children.

## Introduction

Autism spectrum disorder (ASD) is an early-onset, lifelong developmental disorder of neural and mental development (DSM-5/ICD-11). Core features are impairments in social communication already present in childhood and restrictive, repetitive behaviors. The degree of severity of language and cognitive impairments varies, with the majority of affected individuals having below average abilities. Eighty percentage of affected individuals have at least one comorbid disorder that significantly affects the course of symptomatology. The disorder is often associated with a significantly reduced quality of life as well as high family burden.

Underlying the diverse pathology and heterogeneity of ASD is a complex interaction of genetics, environmental exposures and systemic pathophysiologies, all of which have shown to impact brain connectivity and information processing (Holiga et al., 2019; Cheroni et al., 2020; Carroll et al., 2021; Panisi et al., 2021).

ILF neurofeedback has recently been shown to produce significant changes in functional connectivity in multiple areas and neuronal networks of the brain (Dobrushina et al., 2020). Using this neurofeedback variant for the treatment of ASD is therefore reasonable, especially given that its methodological development has been based on clinical findings (Othmer et al., 2011) and that it is included in evidence-based treatment recommendations for many mental disorders. The intention of this case study is to demonstrate the efficacy of ILF neurofeedback in autism while highlighting the need for further clinical research of the associated brain physiological changes.

## Methods

### Clinical Case

This is a report of the procedures and impacts of a six-month course of clinical ILF neurofeedback treatment on a 5-year-old boy “E” with ASD, who had received his diagnosis at 2.7 years of age on the basis of several assessment instruments: “Diagnostic Interview for Autism (ADI-R),” “Diagnostic Observation Scale for Autistic Disorders (ADOS 2, Modul 1),” and “Social Communication Questionnaire” (“Fragebogen zur Sozialen Kommunikation (FSK)**—**Eltern”).

### ILF Neurofeedback Methods

The origins of neurofeedback, measuring brain waves as electroencephalogram (EEG), decomposing them into their frequency components (“frequency bands”), and then converting their amplitudes into audio-visual feedback signals, go back to the 1970s (Sterman, 1973). ILF neurofeedback was primarily developed empirically, based on clinical observations from “frequency band” training protocols and those neurofeedback methods that utilized slow cortical potentials (SCP) in the EEG frequency range below 0.1 Hz, the so-called ILF range (Othmer and Othmer, 2016). The applied treatment protocol of ILF neurofeedback captures in a continuously recorded full-band EEG both the time course of the surface potential in the ILF range and supra threshold power densities of nine discrete frequency bands in the 0.5–40 Hz spectral range and processes all of this into audio-visual feedback signals for the patient (Legarda et al., 2011; Othmer et al., 2011; Othmer, 2015; Othmer and Othmer, 2016; Grin-Yatsenko et al., 2018).

The thresholds of the nine frequency bands adjust individually and dynamically so that the prevailing EEG power density of a frequency band is subthreshold about 95% of the time. Due to this method, a sudden increase of the power density in any of the nine frequency bands immediately leads to suprathreshold values and thus to a strengthening of a certain set of audio-visual feedback signals (“Inhibits”). At the same time the dynamics in the ILF range are tracked as well (“Training signal”) and coupled to a second set of audio-visual feedback signals (“Signal”). For determination of the ILF component the therapist has to set an individual amplification factor *via* a lowpass filter cutoff frequency. The ILF neurofeedback protocol furthermore specifies a bipolar montage for the EEG recording. Thus, it is not the dynamically changing brain activity underneath each two electrodes, but rather their difference, that is the targeted signal; and consequently, ILF neurofeedback targets network relationships directly, and accordingly represents a coherence training.

### 10/20 Electrode Position Nomenclature

In this study, we follow the standard 10/20 electrode position nomenclature of the American Clinical Neurophysiology Society. This nomenclature underwent some changes in its last update in 2016, which also affect some of the electrode positions commonly used in ILF neurofeedback. Since this journal often still uses the old designations, we would like to point out that the actual “T8” placement corresponds to the old “T4” terminology.

### EEG Recording System and Montage

The clinical neurofeedback utilized the ILF neurofeedback protocol developed by Othmer et al. (2011), Othmer and Othmer (2016), Othmer (2015) and instrumented by the Cygnet system, which consisted of a 2-channel differential “NeuroAmp II” EEG amplifier (Corscience, Germany), as well as and Cygnet software (BEE Medic, Germany). This system integrates with video animation feedback (Somatic Vision, USA), runs on computer with Windows 10 operating system, and uses an additional high-resolution monitor to display the video animations.

Each neurofeedback session consisted of a continuous differential 2-channel full-band EEG recording that was carried out using a bipolar electrode montage at T8-P4 placement, with an additional electrode at Cz for reference (of both channels) and a grounding electrode at Fpz. Before placing electrodes, the skin at the placement area was cleaned by abrasive paste (Nuprep, Weaver and Company, USA). Then Ten20 Conductive Paste (Weaver and Company, USA) was used to hold the electrodes in place and to ensure low impedances (<5 kΩ) of all electrodes.

### Symptom Tracking

To assess symptom changes through ILF neurofeedback therapy, we used the online symptom tracking tool of EEG Expert (www.eegexpert.net), which has as its central element a catalog of 137 symptoms from the categories of sleep, attention and learning behavior, sensory and perception, behavior, emotions, physical symptoms and pain. For an initial survey the severity of each of the 137 given symptom has to be rated according to a scale ranging from 0 (symptom does not apply at all) to 10 (symptom occurs very frequently or is maximally pronounced). To track symptom changes during and after the end of the clinical intervention the therapist can select up to 25 symptoms, for which a particularly high severity was indicated in the initial survey or for which there is a particular clinical interest, for further surveys.

The parents of “E” were asked to track the individual symptoms of her son before (survey 1 on 16 October 2018) and after (survey 2 on 30 April 2019) the neurofeedback intervention. Between the two points of measurement was the phase of 26 sessions of ILF neurofeedback intervention. After the initial assessment of symptom severities, a total of 23 symptoms with the highest severity levels were selected for the second survey. Table 1 shows the 23 symptoms selected in this step with the evaluated severity levels at the two time points of the surveys.

**Table 1:** List of all tracked symptoms of “E” with course of severity before the beginning (survey 1, 16 October 2018) and after (severity 2, 30 April 2019) the end of the intervention with ILF neurofeedback.

**Category**	**Symptom**	**Severity pre-interv. (survey 1)**	**Severity post-interv. (survey 2)**	**Severity reduction [%]**
Sleep	Difficulty maintaining sleep	10	2	80
Sleep	Night sweats	10	0	100
Sleep	Restless sleep	10	1	90
Sleep	Bruxism	10	1	90
Sleep	Difficulty falling asleep	10	0	100
Sleep	Nocturnal enuresis	10	10	0
Attention/Learning	Poor drawing ability	10	8	20
Attention/Learning	Difficulty shifting attention	10	5	50
Attention/Learning	Poor verbal expression	10	4	60
Sensory	Auditory hypersensitivity	10	3	70
Sensory	Poor body awareness	10	7	30
Sensory	Somatosensory deficits	10	5	50
Sensory	Poor balance	9	0	100
Sensory	Clumsiness	10	4	60
Behavioral	Excessive talking	10	10	0
Behavioral	Impulsivity	10	10	0
Behavioral	Self-injurious behavior	10	8	20
Behavioral	Autistic stimming	10	8	20
Behavioral	Hyperactivity	9	7	22
Behavioral	Oppositional/defiant behavior	8	6	25
Emotional	Anxiety	10	6	40
Physical	Fatigue/exhaustion	10	4	60
Physical	Weak immune system	10	0	100
	Σ	226	109	Ø 52

*The 23 symptoms listed here were selected from a list of 137 symptoms in the categories of sleep, attention and learning behaviors, sensory and perception, behavior, emotions, physical symptoms, and pain after the first complete symptom severity survey based on the highest severity levels reported by the parents of “E”*.

### Initial Presentation

In the intake interview the mother of “E” reported that he displayed a lack of social engagement, as in kindergarten, not actively approaching other children to either participate in their activities or to ask them to participate in joint activities.

Additionally, “E” did not play on his own, did not engage in pretend play games, and did not imitate people's behavior; but he had already started to imitate behavior from cartoons. Moreover, “E” had difficulties with expressing his feelings and maintaining eye-contact with other people. In addition, when he was either feeling excited or tired he would flap his hands, and engage in self-destructive behavior such as biting his fingers. His mother also reported problems with bedtime and sleeping: “E” struggled with bedtime at evening and had tantrums throughout the night, which lasted about 3–5 h with occasional meltdowns. Further reported symptoms included noise sensitivity, bruxism, incontinence during the day and night, emotional reactivity, fearfulness and clinginess. “E” showed coordination problems and was, for instance, not able to properly use the foot pedals of his play tractor to ride it. Furthermore, he had not hit age-appropriate milestones in his language and speech development; instead, he voiced specific, difficult to understand sounds and repeated them excessively.

### Treatment Sessions 1–12: Initial Approach and Rationale

#### Initial Right-Sided Training at Temporal-Parietal Sites T8-P4

To address the reported symptoms of “E” with ILF neurofeedback, EEG electrodes were placed in the beginning of all training sessions on the right side of his skull at the temporal-parietal sites T8-P4, as this placement is known to calm sensory hypersensitivities, to improve sensory integration and to increase social-emotional awareness and empathy (Othmer, 2015).

At the beginning of the ILF neurofeedback interventions “E” would not allow the therapist to attach electrodes and repeatedly took them off. Therefore, he was rewarded with sugar free sweets for every electrode he allowed the therapist to attach electrodes to his head.

Using this procedure, it became possible to conduct a first session of ILF neurofeedback. “E” watched a short movie (for 12 min) where feedback was given *via* dynamic modulation of the size of the movie window according to the strength of the recorded EEG activity within the (ILF) training signal frequency range.

#### Adjustments Based Upon Clinical Indications

Session two and three were used to adjust the ILF training signal frequency setting in order to find his optimal response frequency (ORF) and to extend the session duration to 15 min. Prior to the next neurofeedback session, his mother reported a noticeable improvement in “E's” sleep performance.

After the fourth session of neurofeedback, “E” again experienced difficulties falling asleep at night and therefore the ILF training signal frequency setting was adjusted in the fifth session.

#### Observed Impacts

By the end of the first session, no remarkable changes in “E” could be observed. However, his mother later reported that about 1 h after the session, she had been able to order food in a McDonald's Drive-Through for the very first time without him yelling and screaming out of fear of the voice from the loudspeaker.

The night after the ORF adjustment in the fifth session of neurofeedback, he slept exceptionally well for 10.5 h without interruptions. According to the reports of his parents, also “E's” mood in the mornings had improved to a remarkable degree and he had started waking up feeling happy and relaxed.

In parallel to the improvements in sleep, “E” managed for the first time to properly use the foot pedals and steering wheel of his child tractor, and thus was able to drive the tractor correctly. Additionally, he newly added the new word “Tante” (German for “aunt”) to his vocabulary.

After eight sessions of neurofeedback, “E” began to engage in role playing. In one specific imaginary scenario he imagined himself in the role of the therapist and his client was his teddy bear. Moreover, he imitated the entire neurofeedback training session procedure on his teddy bear, including insisting on placing electrodes on the teddy bear's head. He even rewarded the teddy bear with sweets after the placement of each EEG electrode.

### Treatment Sessions 13–26: Electrode Placements at T8-P4 and T8-F8

To target his developmental speech delay and to trigger speech production, a second training with right frontal-temporal electrode placements at T8-F8 for 15 min was added from the 13th session on directly after 15 min of right parietal training at T8-P4.

#### Observed Impacts

“E” produced 33 new words such as “Bärli,” “Mona,” “Romi,” etc. Additionally, after the 26th session he produced the four-word sentence: “Papa ist zu Hause” (German for “Dad is at home”).

The mother of “E” reported the following changes in her son during the full course of the ILF neurofeedback training (16 October 2018–30 April 2019): “*The hand-flapping decreased and now solely occurs in really exciting moments, e.g., when watching a movie for the first time or when he is with many friends or other people. Moreover, he dramatically hit speech milestones and became able to produce three-to-four-word sentences, which mostly consisted of nouns, but also slowly started to involve verbs*.

*It was also noticeable that he started to sing songs again (which he had stopped when he was about two years old) and was getting more and more aware of the meaning of the lyrics. Furthermore, he started to clearly adhere to rules and to prefer to do everything on his own. Moreover, he became able to tell us verbally or through gestures which part of his body was hurting. In my perception, the changes achieved in my son through neurofeedback training are clear and significant*.”

### End of Treatment

After “E's” parents felt that the goals of ILF neurofeedback therapy had been achieved, they decided to discontinue treatment after the 26th session.

## Results

After the first symptom severity survey by the parents of “E” before the start of the NFB intervention, the symptoms with the highest evaluation scores were selected for re-evaluation after the end of the treatment. The result is shown in Table 1 and indicates that some symptoms with initial maximum evaluated severity, like *night sweats, difficulty falling asleep* and others, had completely disappeared during the course of the intervention with ILF neurofeedback. However, the evaluated severity of the symptoms *nocturnal enuresis, impulsivity* and *excessive talking*, were not affected by the intervention at all. The overall reduction in severity of the 23 selected symptoms due to the ILF neurofeedback intervention was 52%.

**Figure 1:**
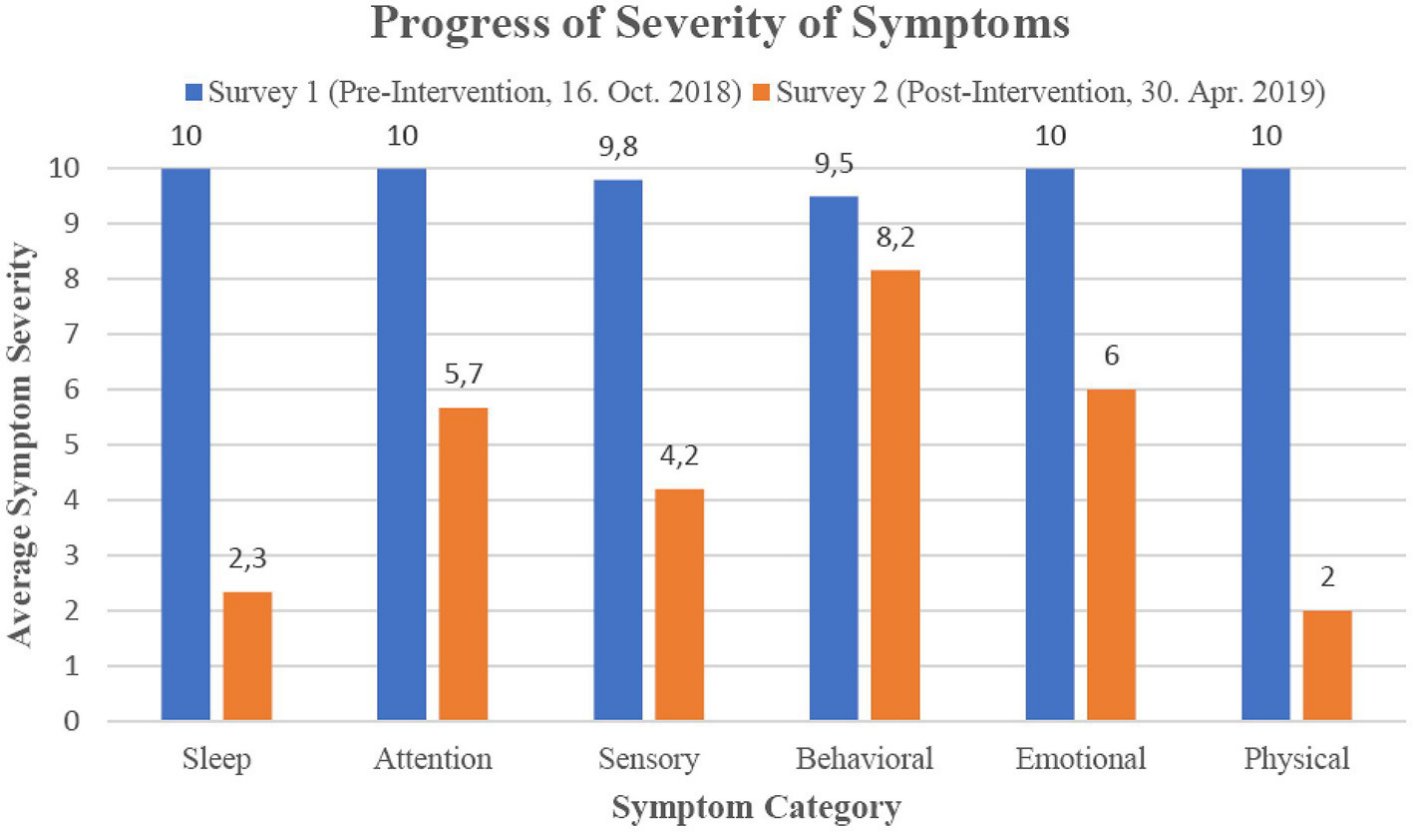
Progression of percent reduction in categorized symptoms by ILF neurofeedback intervention.

To determine which areas show the greatest symptom changes, the 23 individual symptoms that had the largest scores in the initial survey were grouped into the categories *Sleep, Attention*/*Learning, Sensory, Behavioral, Emotional* and *Physical* (see Table 2, Figure 1). The ILF neurofeedback training decreased the average symptom severity of every category to a remarkable degree, with the strongest effect (80% and 77% mean severity reduction) for physical and sleep symptoms and the lowest influence (15% mean severity reduction) on behavioral symptoms.

**Table 2:** Aggregate severity reduction for the entire symptom group over the course of 26 neurofeedback sessions.

**Symptom category**	**Severity reduction [%]**
Sleep	77
Attention, learning	43
Sensory	58
Behavioral	15
Emotional	40
Physical	80
Aggregate severity	52

## Discussion

In the case of “E,” the ILF neurofeedback intervention of 26 sessions over a time period of 6.5 months at T8-P4 electrode training sites and at the individual optimal response frequency within the ILF range below 0.1 Hz, clearly had led to general physical calming and a reduction of his arousal level as well as to an improvement of flexibility and stability of state regulation. In consequence, most of his sleeping problems had improved dramatically over the intervention period. Distinct improvements were also recorded in the areas of social behavior and body coordination over the course of the intervention.

After a more frontal electrode position at T8-F8 was added in the second half of the training, “E” showed remarkable improvements in his body perception and speech development. Comparable symptom changes in language development after ILF neurofeedback intervention have been reported in a case description of a 4-year-old child with ASD (Sasu, 2020). The restriction to a purely right-hemispheric electrode placement from the 13th neurofeedback session onwards to promote the linguistic development of “E” has its rationale in the empirical finding that such training often is the key to the emergence of language in ASD children (Othmer and Othmer, 2011). This group of patients with early childhood developmental deficits or sensory filtering problems usually benefits from right side ILF neurofeedback training, which is consistently demonstrated by improvements in brain functions that are distant from the electrode placements or localized in the left hemisphere. Thus, “E” also shows a symptom severity improvement of 60% with respect to his linguistic expression by a purely right-hemispheric electrode placement, even though language production and also language-specific networks are generally localized in the left hemisphere. It should be noted, however, that the cortical structures, whose EEG activities are converted into audio-visual feedback signals during ILF neurofeedback, do not necessarily represent the brain areas that are affected by the training. Consistently, in a controlled fMRI study with 53 healthy subjects, Dobrushina and colleagues show after purely right-sided ILF neurofeedback training at electrode sites T4-P4 significantly increased connectivity on both hemispheres between salience, language, and visual networks, particularly including portions of Broca's area and Wernicke's region (Dobrushina et al., 2020). We conclude that this case description cannot provide a more detailed explanation for the interesting finding of improved linguistic expression after right-sided ILF neurofeedback treatment and further clinical research is required.

The aforementioned changes in severity are also evident for many other symptoms of “E,” as revealed by the two symptom severity surveys before the ILF neurofeedback intervention began and after it ended. The symptoms initially rated as most severe were alleviated by 52% after the course of neurofeedback. A grouping of the symptoms revealed that “E” was able to benefit from the neurofeedback training especially in the category of *physical* and *sleep* symptoms (80 and 77%, respectively). In comparison, the change in severity of behavioral symptoms during the neurofeedback intervention was much smaller (15%), although these also decreased. This is an interesting finding because the clinical literature on the effects of neurofeedback in ASD tends to conclude that this method of treatment addresses ADHD-specific symptoms, such as *hyperactivity* or *impulse control* (for review see Holtmann et al., 2011). But is has to be mentioned that protocols distinctly different from ILF neurofeedback were used in these studies. However, a study of the effect of ILF neurofeedback in children and adolescents with ADHD shows significant improvements in ADHD-specific symptoms (Schneider et al., 2021), which demonstrates that this neurofeedback method in principle can affect symptoms of hyperactivity and impulse control. In addition, the results presented must be considered in light of the limitations of this study, as it represents only a single case description and the variability of results is particularly high in autism, in our experience. Thus, further clinical and controlled studies are needed to clarify the particulars of ILF neurofeedback treatment effects on behavior-specific symptoms in children with ASD.

## Conclusion

The results presented on the intervention in a child with ASD indicate that ILF neurofeedback is a suitable clinical training method for addressing a diverse range of autistic behaviors characteristic of this spectrum disorder in children, and that it can help to reduce their developmental deficits through enhanced self-regulation of the central nervous system.

Given the degree of progress achieved in 26 sessions of ILF neurofeedback intervention over 6 months, the method appears to be cost-effective as well as clinically effective. It also directly addresses connectivity mechanisms central to ASD, thus laying the basis for further improvements with maturation. Therefore, ILF neurofeedback deserves further investigation, including case studies and controlled clinical studies addressing protocol particulars and underlying mechanisms, with the aim of laying the foundation for broader implementation.

## Data Availability Statement

The original contributions presented in the study are included in the article/supplementary material, further inquiries can be directed to the corresponding author/s.

## Author Contributions

AR: therapist of E and co-author. HS: responsible for the technical part in the case study and co-author. WP: responsible for the pedopsychiatric aspects in the case study and co-author. All authors contributed to the article and approved the submitted version.

## Conflict of Interest

HS was employed by BEE Medic Gmbh Germany. The remaining authors declare that the research was conducted in the absence of any commercial or financial relationships that could be construed as a potential conflict of interest.

## Publisher's Note

All claims expressed in this article are solely those of the authors and do not necessarily represent those of their affiliated organizations, or those of the publisher, the editors and the reviewers. Any product that may be evaluated in this article, or claim that may be made by its manufacturer, is not guaranteed or endorsed by the publisher.
